# Supplementary material for: Characterization of the urinary DNA virome of hematopoietic stem cell transplant recipient and healthy cynomolgus macaques
Source: bioRxiv. 2026 May 6:2026.05.05.722665. Preprint. [Version 1] doi: 10.64898/2026.05.05.722665 (PMC13174661; doi:10.64898/2026.05.05.722665)
Supplement: Supplement 1 — Fig. S1. Partial amino acid alignment of Large T antigen between previously isolated samples and MafaPyV2 and SV40 type IIB. [file media-1.pdf]

Fig. S1

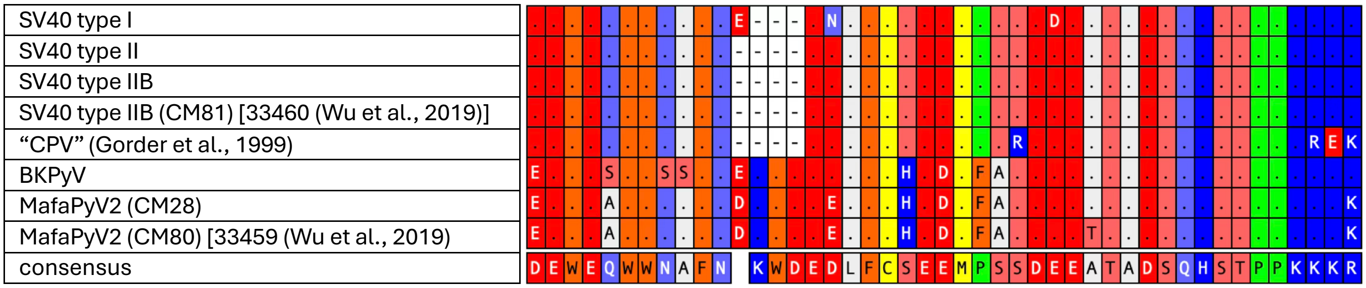

SV40 type I . . . . E - - - . N . . . . D . . . .
SV40 type II . . . . - - - - . . . .
SV40 type IIB . . . . - - - - . . . .
SV40 type IIB (CM81) [33460 (Wu et al., 2019)] . . . . - - - - . . . .
“CPV” (Gorder et al., 1999) . . . . - - - - . . . . R . . . . R E K
BKPyV E . . . S . . S S . . E . . . . H . D . F A . . . .
MafaPyV2 (CM28) E . . . A . . . . . . D . . . . E . . . H . D . F A . . . . K
MafaPyV2 (CM80) [33459 (Wu et al., 2019) E . . . A . . . . . . D . . . . E . . . H . D . F A . . . . T . . . . K
consensus D E W E Q W W N A F N K W D E D L F C S E E M P S S D E E A T A D S Q H S T P P K K K R
