# Supplementary figures and images for: Characterization of the urinary DNA virome of hematopoietic stem cell transplant recipient and healthy cynomolgus macaques
Source: bioRxiv. 2026 May 6:2026.05.05.722665. Preprint. [Version 1] doi: 10.64898/2026.05.05.722665 (PMC13174661; doi:10.64898/2026.05.05.722665)

Fig. S2

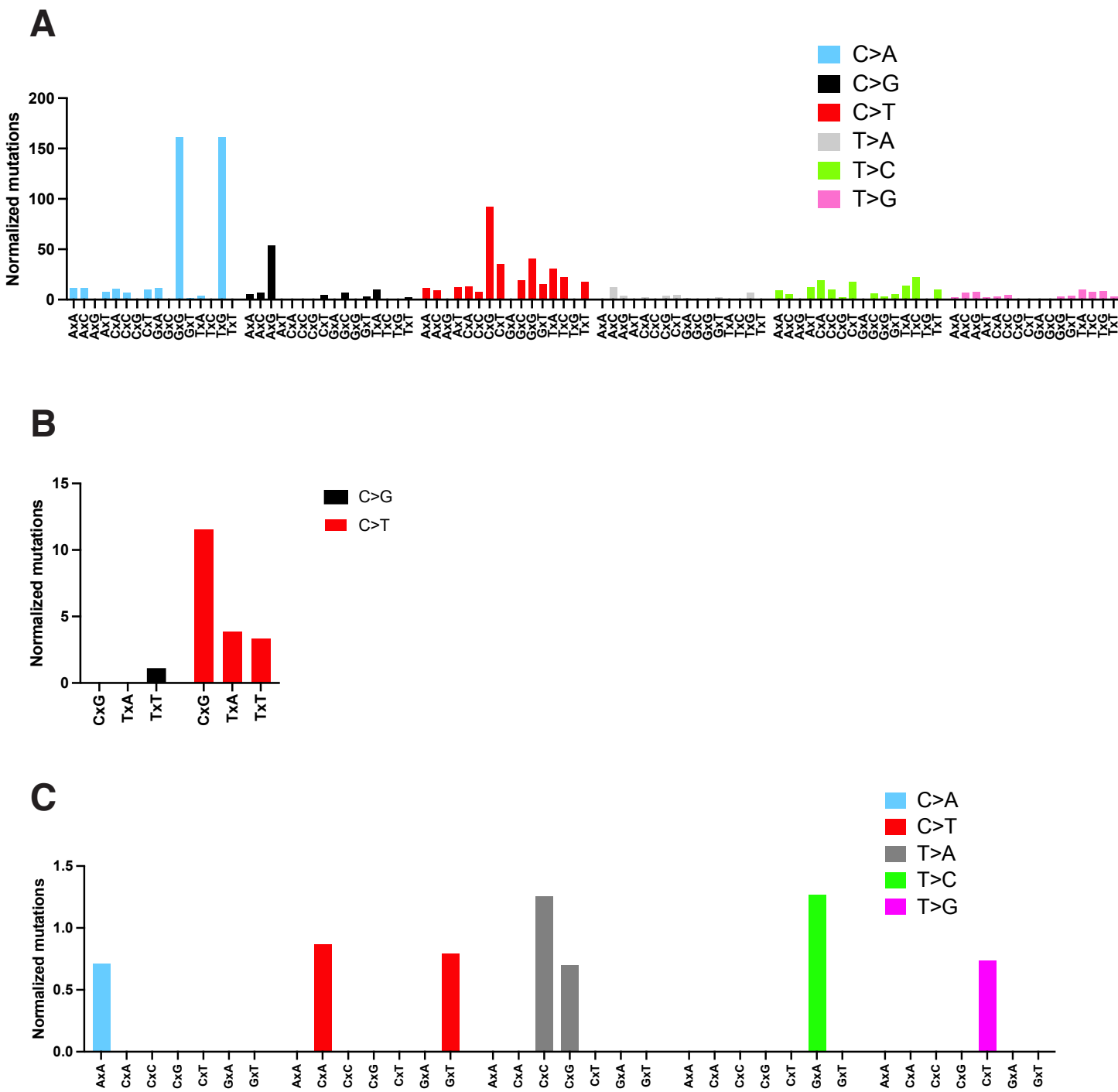

Supplement: Supplement 2 — Fig. S2. Normalized intra-host mutational signatures of A) MafaPyV2, B) MafaPyV3, and C) SV40 type IIB. [file media-2.pdf]

Fig. S3

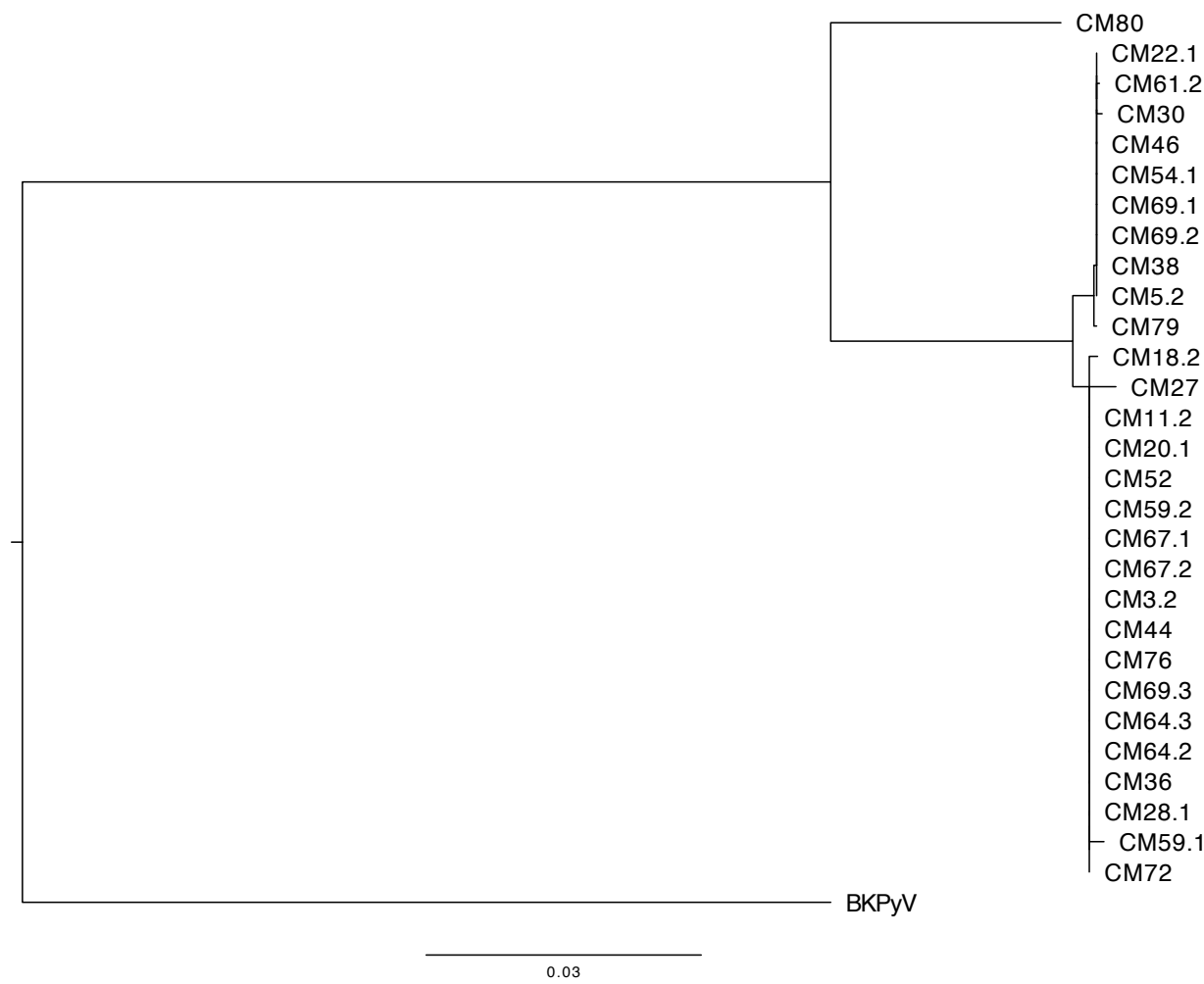

Supplement: Supplement 3 — Fig. S3. Phylogenetic tree of MafaPyV2 complete genomes with at least 90% coverage. [file media-3.pdf]
